# Multimodal Treatment of a Complicated Esophagopleural Fistula After Boerhaave’s Syndrome: Successful Resolution With Endoscopic Vacuum Therapy

**DOI:** 10.7759/cureus.96613

**Published:** 2025-11-11

**Authors:** Luiza A Martinez, Carolina B Graciolli Facanali, Marcelo R Borba, Jose Luiz A Gonçalves, Felipe F Malta, Carlos W Sobrado, Marcio R Facanali

**Affiliations:** 1 Medicine, Universidade do Oeste Paulista, Guarujá, BRA; 2 Gastroenterology, Hospital das Clinicas da Faculdade de Medicina da Universidade de Sao Paulo, Sao Paulo, BRA; 3 Gastroenterology, Hospital das Clínicas da Faculdade de Medicina da Universidade de Sao Paulo, Sao Paulo, BRA; 4 Gastroenterology, Universidade do Oeste Paulista, Guarujá, BRA; 5 Intensive Care Unit, Hospital Frei Galvão, Santos, BRA; 6 Gastroenterology, Hospital das Clínicas da Faculdade de Medicina da Universidade de São Paulo, Sao Paulo, BRA

**Keywords:** boerhaave’s syndrome, empyema, endoscopy, esophageal fistula, esophagopleural fistula, gastrointestinal, negative-pressure wound therapy, pleural

## Abstract

Esophagopleural fistulas are rare complications associated with high morbidity and mortality, often resulting from spontaneous or iatrogenic esophageal perforations. We report a case of a 60-year-old male presenting with acute respiratory failure and empyema after spontaneous esophageal rupture (Boerhaave’s syndrome). Despite thoracotomy with pleural decortication, surgical closure of the fistula was not feasible due to its proximity to the descending aorta. We report a technically complex case of esophagopleural fistula located near the descending aorta, in which delayed diagnosis and extensive inflammatory reaction obscured the tissue planes, precluding safe surgical dissection and repair. Endoscopic vacuum therapy (EVT) was therefore employed as a successful rescue strategy. A multimodal approach was adopted, including intensive care, broad-spectrum antibiotics, drainage procedures, and subsequent EVT. EVT was performed using a handmade sponge system constructed with gauze attached to a nasogastric tube, initially positioned intracavitarily, and exchanges were performed every seven days. After 14 days of EVT, complete closure of the fistula was confirmed endoscopically and radiologically. This case highlights EVT as an effective rescue strategy for complex esophagopleural fistulas, underscoring the importance of individualized, multidisciplinary management.

## Introduction

Boerhaave’s syndrome, described in 1724, corresponds to a spontaneous transmural esophageal rupture, usually precipitated by forceful vomiting, leading to mediastinal contamination and pleural extension that may evolve into esophagopleural fistula (EPF). Despite its rarity, with an incidence of approximately 3.1 cases per million annually, it remains one of the most lethal gastrointestinal emergencies, with mortality rates exceeding 40% [1,2]. Clinical presentation is often nonspecific, and the classic Mackler’s triad (vomiting, chest pain, subcutaneous emphysema) is present in fewer than half of cases, leading to diagnostic delay [3].

EPF represents a severe complication of Boerhaave’s syndrome. Traditional management strategies include surgical repair, conservative treatment, or endoscopic interventions such as self-expanding stent placement [4,5]; however, these approaches may be limited by stent migration, incomplete sealing, or persistent leakage, which underscores the potential value of endoscopic vacuum therapy (EVT) as a rescue option. Recently, EVT has emerged as a promising alternative, offering continuous drainage, enhanced granulation, and high closure rates, reported between 80% and 90% in gastrointestinal transmural defects [6-9]. We present a case of complicated EPF successfully managed by multimodal treatment culminating in EVT.

## Case presentation

**Figure 1:**
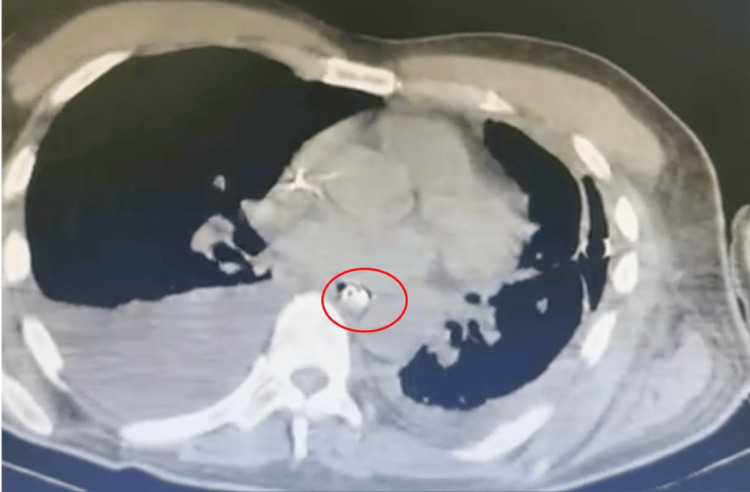
Bilateral pleural effusion and esophageal vacuum therapy catheter on CT. Chest computed tomography showing bilateral pleural effusion and an intraluminal esophageal catheter for vacuum therapy (red circle).

A 60-year-old man with a history of asthma and chronic nephropathy presented to the emergency department with severe epigastric pain and progressive dyspnea following spontaneous, forceful vomiting, with no antecedent alcohol intake or other triggering factors. He rapidly deteriorated, requiring intubation for acute respiratory failure. Initial CT scan revealed a large left pleural effusion and mild mediastinal air, without clear evidence of esophageal perforation; however, these findings raised suspicion for a transmural defect consistent with Boerhaave’s syndrome. Thoracic drainage yielded purulent fluid consistent with empyema (Figure 1).

**Figure 2:**
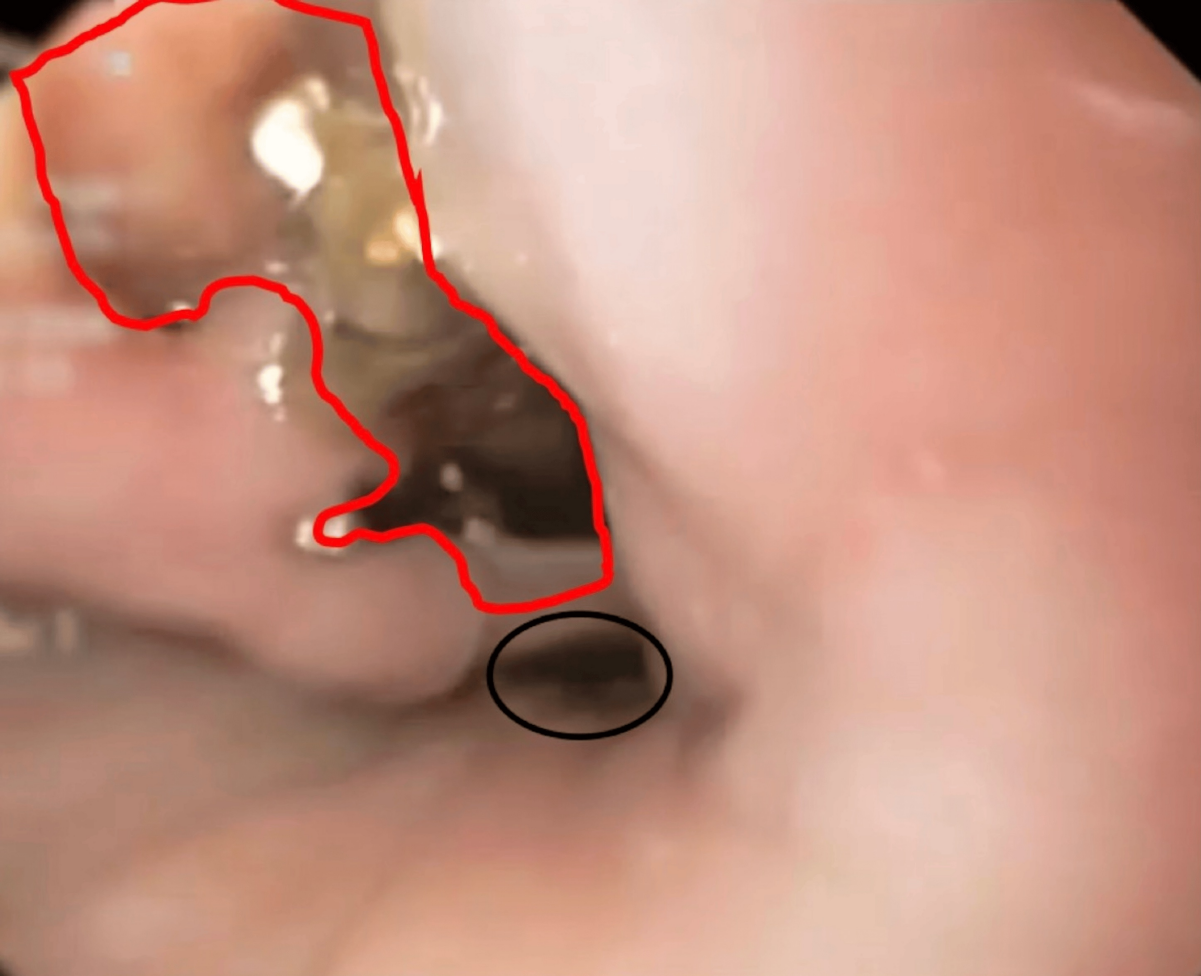
Upper endoscopy showing distal esophageal perforation near the esophagogastric junction. Upper endoscopy showing a distal esophageal perforation/laceration (red line) near the esophagogastric junction (black circle).

During hospitalization, enteral feeding leakage through the chest tube was observed. On the third day after admission, while on nasoenteral feeding, prior to EVT, leakage through the chest tube was observed, indicating a pleuroesophageal communication. Methylene blue testing and upper endoscopy confirmed a distal esophageal perforation with a fistulous tract located approximately 38 cm from the incisors, just above the gastroesophageal junction, covered by fibrin and purulent secretions (Figure 2). Broad-spectrum antibiotics were initiated, and pleural drainage, previously placed upon admission, was maintained for infection control. Despite intensive care, the patient developed septic shock, requiring transfer to a tertiary ICU.

On day 20, a left posterolateral thoracotomy was performed, revealing multiple adhesions, purulent secretion with food debris, and a 2 cm fistula firmly adherent to the descending aorta. Primary surgical repair was deemed unsafe, and pleural decortication with drainage was performed.

**Figure 3:**
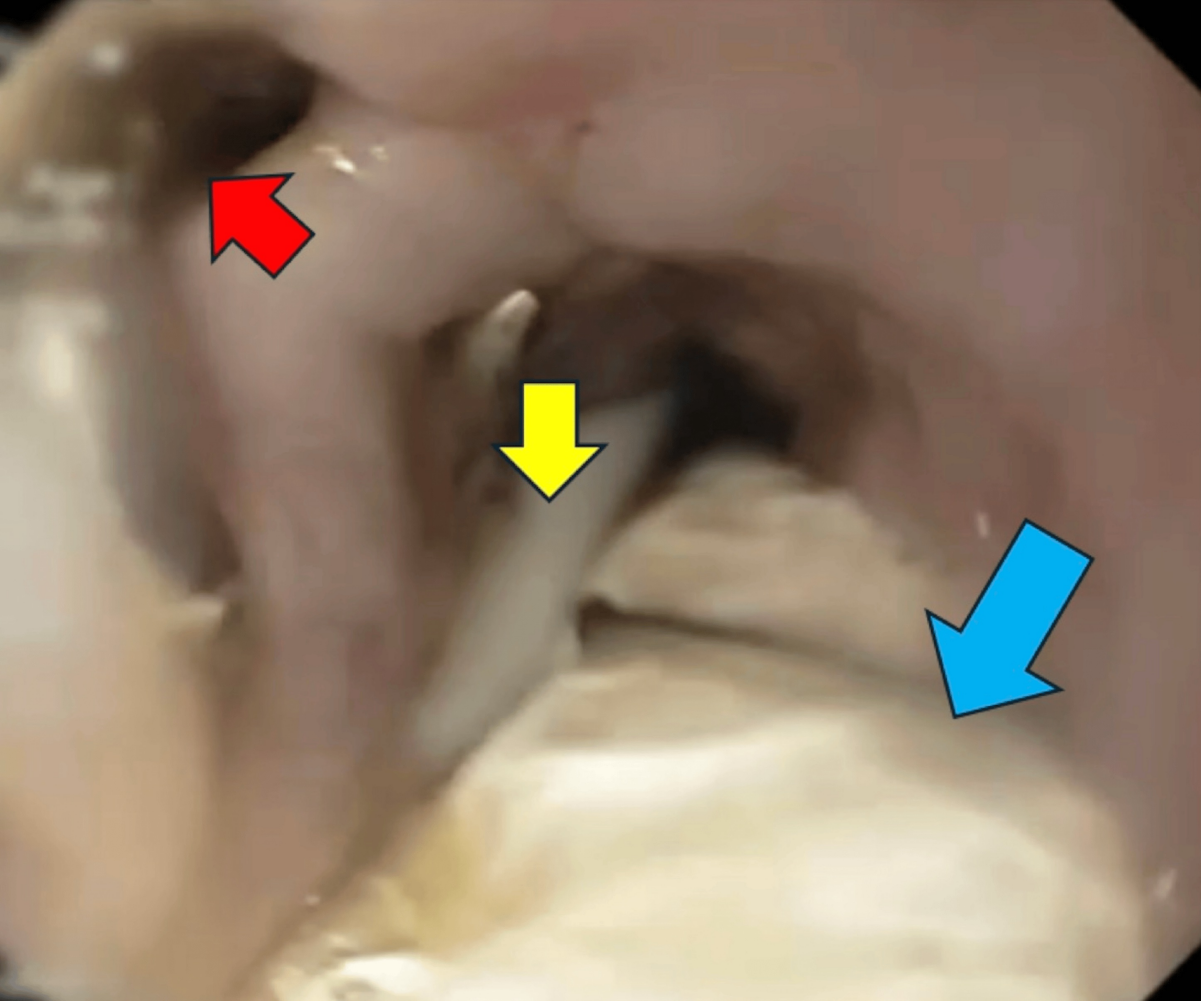
Upper endoscopy showing esophagopleural fistula orifice, nasoenteral tube, and vacuum therapy catheter. Upper endoscopy of the distal esophagus showing the esophagopleural fistula orifice (red arrow), the nasoenteral tube (yellow arrow), and the vacuum therapy catheter (blue arrow).

Despite thoracotomy and continued drainage, sepsis persisted with ongoing purulent output through the chest tube, leading to the decision to initiate EVT on day 27 as a rescue measure. An adapted system was assembled using a 16-Fr nasogastric tube wrapped with gauze and covered by a plastic film, connected directly to the hospital wall vacuum. A 20-gauge needle was used to perforate the circuit, ensuring adequate negative pressure. The device was positioned endoscopically at the level of the fistula in an intraluminal position (Figure 3), while a feeding tube was advanced beyond the ligament of Treitz for enteral nutrition.

**Figure 4:**
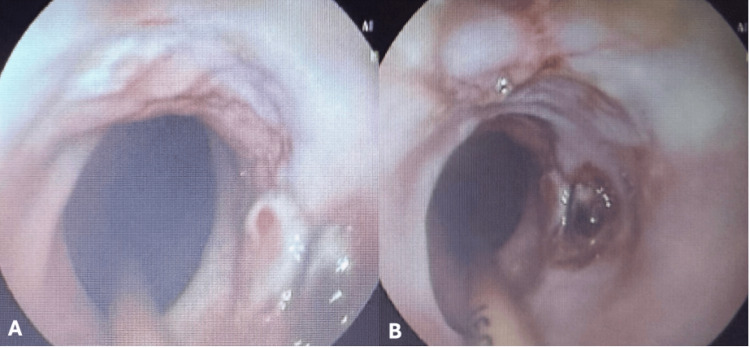
Upper endoscopy demonstrating complete healing of the esophagopleural fistula. Upper endoscopy showing complete closure of the esophagopleural fistula after 14 days of treatment with a vacuum therapy catheter.

EVT was maintained for 14 days, with two exchanges performed every seven days, consistent with our institutional protocol to balance procedural safety and effective drainage. After 14 days of EVT, follow-up endoscopy demonstrated complete closure of the EPF (Figure 4), confirmed by contrast-enhanced CT. The healed area showed mild luminal narrowing but allowed smooth passage of a 9.8-mm endoscope, with mature, non-friable granulation tissue and no stricture formation. The patient improved progressively, chest drains were removed, oral intake was resumed, and he was successfully discharged from the intensive care unit. After an additional 18 days of hospitalization, with gradual weaning from parenteral nutrition and transition to exclusive oral feeding, the patient was discharged in good general condition and remains under follow-up with gastroenterology and thoracic surgery teams, with a new upper endoscopy scheduled in six months.

## Discussion

Spontaneous esophageal rupture remains a diagnostic and therapeutic challenge. Delayed recognition, as in this case, significantly increases morbidity and mortality, particularly when complicated by EPF [1-3]. Early surgical repair within 24 hours is associated with improved survival, but delayed intervention often necessitates complex multimodal strategies [4,5].

Minimally invasive endoscopic techniques, especially EVT, have shown high success rates (>80%) and lower mortality compared with conventional approaches [6,7]. In our case, EVT was performed with an adapted system assembled from commonly available hospital materials, which achieved effective drainage and tissue healing. This illustrates the feasibility of EVT even outside commercially available sponge systems, without compromising therapeutic efficacy.

Other reports have also demonstrated the efficacy of EVT for EPFs, including pediatric and eosinophilic esophagitis-related cases, supporting its expanding role across different contexts [10,11].

In our institution, a gauze-based configuration was preferred over a polyurethane sponge or dual-tube system due to its softer texture and easier assembly, reducing the risk of mechanical trauma to friable esophageal tissue. Although sponge-based systems provide more uniform suction, they require specific materials not routinely available in our setting. The gauze configuration ensured adequate drainage and cavity collapse while maintaining procedural simplicity. EVT exchanges were performed every seven days, consistent with our institutional protocol, balancing anesthesia exposure and drainage efficacy. No fluoroscopic or contrast-guided assessment of the fistulous tract was performed prior to EVT placement, as direct endoscopic visualization and prior surgical exploration had already delineated a well-defined 2-cm esophagopleural communication adjacent to the descending aorta, making additional imaging unnecessary and in line with the European Society of Gastrointestinal Endoscopy (ESGE) recommendations for cases with clearly identified trajectories.

The favorable outcome reinforces EVT as a valuable rescue therapy when primary surgical repair is not possible. Integration of surgery, endoscopy, intensive care, and infection control was essential to achieving definitive closure and patient recovery.

Our case illustrates the feasibility of EVT as salvage therapy after failed surgical repair in a high-risk patient. Integration of thoracic surgery, endoscopy, intensive care, infectious diseases, and nutrition was critical to the favorable outcome. This highlights the need for individualized, multidisciplinary management in such complex scenarios.

## Conclusions

Complicated EPFs after Boerhaave’s syndrome require tailored multidisciplinary strategies. EVT proved to be a safe, effective, and minimally invasive rescue therapy, achieving definitive closure where surgical repair was not feasible. Wider dissemination of EVT techniques may contribute to improved prognosis in similar life-threatening conditions.
